# Caring load and family caregivers’ burden in China: the mediating effects of social support and social exclusion

**DOI:** 10.3389/fpubh.2023.1194774

**Published:** 2023-09-22

**Authors:** Hongwei Hu, Xinyi Hu, Yang Xu

**Affiliations:** ^1^School of Public Administration and Policy, Renmin University of China, Beijing, China; ^2^School of Sociology, Beijing Normal University, Beijing, China

**Keywords:** psychological stress, family caregivers’ burden, older adults, social support, social exclusion, China

## Abstract

**Objective:**

Caring for older adults with disabilities is a source of stress for family caregivers, and the lack of social support and the pressure of social exclusion might aggravate family caregiver burden. This study aimed to examine the association between caring load and family caregivers’ burden, as well as the mediating effects of social support and social exclusion.

**Methods:**

Data used in this study was derived from the nationally representative database of the aged population in China, and 3,125 households with disabled old adults and their home-bound caregivers were eventually selected for this analysis. Regression methods and mediation analysis methods were employed in this study.

**Results:**

The results indicated that there was a significant positive association between caring load and the caregiver burden, and specifically, social support intensity (rather than social support breadth) and passive social exclusion (rather than active social exclusion) played partial mediating effects. Furthermore, the contributions of mediating effects of social support intensity and passive social exclusion were 13–15 and 27–29%, respectively, and the total contribution of mediating effects was about 35–38%.

**Conclusion:**

Family caregivers’ burden should be paid more attention to in the large population with rapidly aging speed like China, and more guidance services as well as support should be provided to family caregivers. In addition, it is crucial to focus on the community’s social support and social exclusion in public policy innovation.

## 1. Introduction

With the improvement of life quality and the extension of life expectancy, population aging has become a worldwide tendency. China’s aging population is large in scale and fast in growth, and the number of older adults with disabilities is enormous. By the end of 2020, there were 249 million people aged over 60 and 167 million people aged over 65. Meanwhile, more than 180 million older adults have chronic diseases, and the disabled or partially disabled older population is as high as 42 million, accounting for 11.8% of the total older population, according to the statistics of the National Health Commission of the People’s Republic of China. World Health Organization defines disability as the loss or limitation of a person’s ability to perform major activities or activities in daily life, which is an important indicator of health (1). In China, according to the Law of the People’s Republic of China on the Protection of Persons with Disabilities, disability is “a person who is mentally, physically, or structurally deprived of a certain kind of tissue or function or is not normal, completely or partially unable to perform a certain activity in a normal manner.” Older adults have become the main component of the disabled population in China, and the quality of life of older adults with disabilities is deficient, and daily life needs care from others.

Family caregivers are responsible for most of the caring service load of older adults with disabilities in most households, especially in China, with a long history of the cultural tradition of filial piety and an imperfect social security system. The burden on family caregivers is severe (2). However, caring for older adults with disabilities is a challenging and complex task, exerting a huge and long-term negative impact on family caregivers. The scores of physiological function, social function and mental health of family caregivers were lower than those of the general population (3). Mental health disorders including insomnia, psychological discomfort, despair, stress, physiological problems, and emotional confusion (anxiety, depression) could be frequently and long-term observed among family caregivers, due to the suffocating workload (including limited social activities, difficulties in work and occupation and less leisure time), which eventually greatly reduced the quality of life of caregivers. In severe cases, family caregivers would withdraw from the care state because of the long-run overload (4).

The research focused on the family caregivers’ burden began in the 1960s, and family caregivers’ burden was regarded as the cost of taking care of patients at that time (5). Caregiver burden was challenging in psychological, physical, social and economic aspects, while caring for family members suffering from disease and disability (6). Caregiver burden is a Negative Care Experience, family caregivers were vulnerable to adverse results from physics, psychologic, economy, society and other aspects, when lacking the support of emotion, information, finance, facilities and others in the process of care, and this would bring about the caregiver burden (7). Caregiver burden could be divided into four aspects, including physical fatigue (including physical pain, disease occurrence), psychological and emotional guilt (depression and anxiety, etc.), economic strain (such as increasing medical expenditure and decreasing occupational competitiveness), and social isolation or helplessness. The patients’ diseases can also disrupt family life and cause adverse events, including family caregivers’ health decline, dysfunction in the family, and an increase in family economic burden (8).

Stress Coping Model Theory was proposed by American psychologist Richard S. Lazarus in the 1960s, and has been widely used in the study of family caregivers’ burden by many scholars in the past decades. Stress research began in the field of physiology and later also focused on the relationship between “life events” and physical and mental diseases. One quantitative research took the lead in studying stress and introducing the concept of “life events,” which were defined as events that affect people’s spirit in life change, as well as the operational definition of stressors (9). There was a very close correlation between an individual’s subjective evaluation of life events and their health. Some research also showed that in real life, it was not those major life events but life disturbances caused by life events that occur all the time around us that affect people’s physical and mental health and bring about undesirable consequences (10). Consequently, caring for older adults with disabilities could be regarded as a “life event” that causes stress to the family caregivers, negatively affecting their physical and mental health.

The Stress Coping Model applied to family caregivers exists in the typical Chinese cultural background-Chinese filial piety, and the introduction of this environmental constraint element of Chinese filial piety strengthens the analytical framework in terms of the inevitability of caregivers’ pressures, as well as the inevitable choice that social network play as the important coping mechanism for stress event. However, family caregivers seem unavoidable in caring for older adults with disabilities in Chinese society with a profound cultural tradition of filial piety. The filial piety culture weakens the possibility of family caregivers “evading” caring for older adults with disabilities. Caring for older adults is because of the tradition of the family care system for older adults, and the family care system for older adults from the filial piety cultural circumstance. China had a tradition of filial piety for thousands of years, for a long-time, family care for older adults has always been in a dominant position in the urban and rural care-system for older adults (11). Therefore, family caregivers are given a typical cultural background in caring for older adults with disabilities. Chinese traditional society is an “Acquaintance society,” which is characterized by a private relationship between people; people interact through this relationship and form a network of relationships. Inconsistent with the traditional Stress Coping Model, we believe that the coping mechanism of social interaction in China maybe has two sides, both positive (social support) and negative (Social exclusion). Coping mechanisms reflect family caregivers’ best efforts to deal with the caring load and their internal needs, and resolve the conflict between them, including evaluating the meaning of care tasks, controlling or changing the care environment, solving or eliminating problems, and alleviating emotional reactions due to care tasks. The outcome of coping mechanisms can affect the life attitudes and concepts, social abilities, and physical and mental health of family caregivers.

Social support refers to the care and support from others (12). It is a general or specific supportive behavior from others, which can improve the individual’s social adaptability and protect them from the adverse environment. Social support plays as a buffer for caregiver burden, and the higher the level of social support is, the lighter the caregiver burden would be (13). One research pointed out that effective social support can enhance the ability to tolerate, cope with and get rid of stressful situations (14). That is, serving as a buffer, social support could alleviate the negative effects of stressors on physical and mental health to maintain and even improve the individuals’ physical and mental health.

Social exclusion was defined as the break between the individual and the whole society (15). Social exclusion was regarded as when individuals or groups were totally or partly excluded from full social participation, according to the participatory nature of social exclusion (16). As family caregivers usually do not have enough time for social interaction and personal development, they not only suffer from the stress in the care process, but also the pressure brought by social exclusion. Social exclusion is the effect of family caregivers on cognition, thus affecting the cognitive evaluation of the excluded on themselves or others. Social exclusion will damage the individual’s self-regulation ability; it will not only damage the health of the excluded, but also lead to bad emotions such as loneliness, jealousy, depression, and anxiety. Therefore, social exclusion will increase the care burden on family caregivers (17).

Based on the Stress Coping Model Theory, “life events” are seen as “stress source.” Whether “life events” can generate stress after acting on individuals mainly depends on two important psychological processes: cognitive evaluation and coping. Coping is the use of behavioral or cognitive approaches to address the needs between the environment and people, and to address the conflict between “life events” and stress. In our study, the life event of caring for older adults with disabilities is a source of stress for family caregivers, which would bring negative impacts on their physical and mental health. Social support and social exclusion are the coping ways of the family caregivers to “life event” or “stress source.” The impacts on the family caregivers’ physical and mental health (caregiver burden) were coping-consequences of “life event” or “stress source.”

**Figure 1:**
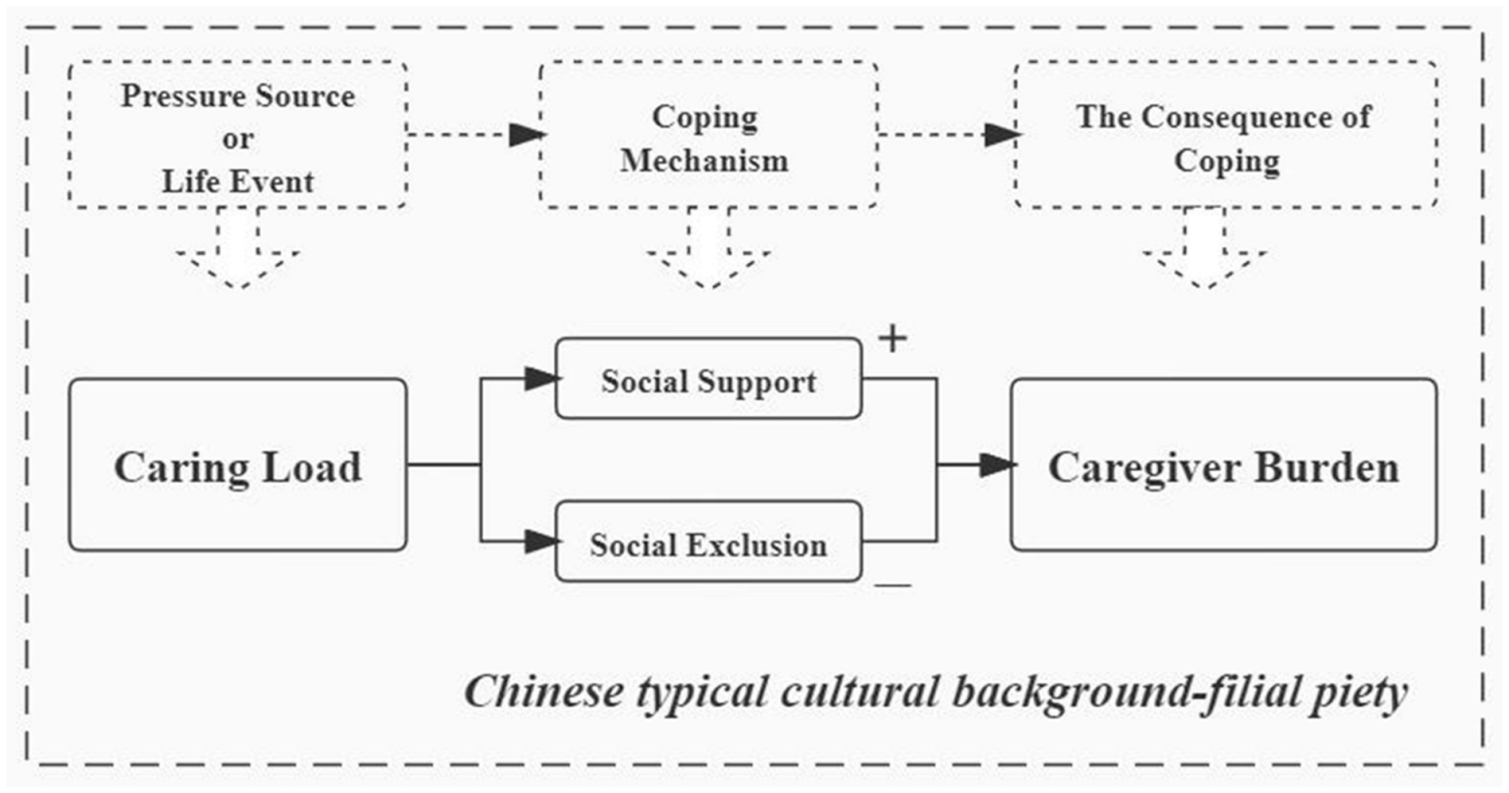
Conceptual framework of mediating mechanism.

Based on the above Stress Coping Model Theory and previous literature, this study proposed the relationship model between caring load and caregiver burden, which included the mediating effects of social support and social exclusion (Figure 1). Furthermore, this study would test the integrated relationship model with a nationally representative database on family caregivers for older adults with disabilities, especially investigating the mediating effects of social support and social exclusion.

This study took the family caregivers of older adults with disabilities as the study population, and made a more comprehensive study on the situation of caregivers’ care burden. On the one hand, it provides a unique experience for reference for the international community to study the care burden of caregivers. Compared with the international community, the research on the care burden of caregivers in Chinese society is insufficient and the research progress is relatively slow. Under the background of the rapid development of aging and the unique long-established filial piety culture in Chinese society, the research on the care burden of family caregivers is more unique and novel, which enriches international literature. On the other hand, problems are identified and policy recommendations are made, thus providing a scientific basis for the government to formulate corresponding policies. Starting with family caregivers, this study explored the influencing mechanism and effective path of caregivers’ care stress, to provide reference and enlightenment for the formulation of related policies to alleviate care stress and improve the quality of care, to improve the quality of life of older adults with disabilities. In addition, the study on the care status of family caregivers is also of great significance for the construction and improvement of China’s long-term care service system.

## 2. Materials and methods

### 2.1. Data

The data used in this study were drawn from Survey on Aged Population in Urban/Rural China (2018), which was conducted from July to September 2018. The survey was approved and sponsored by the Ministry of Civil Affairs of the People’s Republic of China, and carried out by the Institute of Social Science Survey, Peking University. This survey employed a multistage, stratified and random-cluster sampling method, which covered 155 counties (districts) and 1,800 communities (villages) in 28 provinces, autonomous regions, and municipalities in China. Trained investigators conducted home visits. All participants consented to participate in the survey, and the initial sample consisted of 10,273 households. According to the purposes of the study, the study population is caregivers of older adults with disabilities, so we set households screening criteria. Screening households based on two criteria: older adults with disabilities in the household, and older adults with disabilities received care in the household. For households with older adults with disabilities and caregivers, the primary caregivers of older adults with disabilities in the household were asked to complete the survey. After screening, 3,203 households met the above two criteria, of which 78 households had missing values in the corresponding variables and were eliminated from the analyses in this study. Finally, 3,125 households with older adults with disabilities cared by family caregivers were selected and used in this study.

### 2.2. Variables

#### 2.2.1. Dependent variables

Caregiver Burden was measured by the Zarit Burden Interview (ZBI), developed by Zarit et al. The Chinese version of the Zarit Burden Interview has a high level of reliability and validity, with a total Cronbach coefficient of 0.870 (18). It consists of 22 items evaluating five aspects, including caregivers’ health, mental state, economic status, social life and total evaluation. The score of each question ranges from 0 to 4 points, and the total score is from 0 to 88 points. The higher score indicates the heavier caregiver burden.

In the data analysis process, caregiver burden was operated into three variables: burden score, burden grade, self-assessed burden. The burden score is the sum of the scores of 22 items, with a higher score indicating the heavier caregiver burden. According to these references which used the Chinese version of Zarit Burden Interview to measure caregivers’ burden, burden grades consist of little to no burden, light burden, moderate burden and heavy burden, with the range of 0–19, 20–39, 40–59, and 60–88, respectively (19–21). The self-assessed burden was measured by responding to the last question of the scale, “In general, how do you evaluate your caregiver burden?” Self-assessed burden ranges from 0 to 4 points, with 4 indicating severity.

#### 2.2.2. Independent variables

The number of home-bound older adults with disabilities who need care affects the load of family caregivers. In this study, the caring load was measured by the number of older adults with disabilities who need care in households, which is based on the question, “How many disabled older adults were in need of care in your family?”

#### 2.2.3. Control variables

Based on previous studies, three dimensions of factors including individual, family and surrounding characteristics were primary indicators being proved to be associated with Caregiver Burden (22). Individual characteristics included gender, age, marital status and education status. Family characteristics included family size and household income. Surrounding characteristics were measured by the number of social service supplies in the community, including meal services, bath services, housekeeping services, daycare services, rehabilitation care services, health education services, psychological counseling services, home medical services, medical care services, social work services and respite services.

#### 2.2.4. Mediating variables

Social support is the state that a person obtains through social contact, which could reduce psychological stress, relieve tension, and improve social adaptability. The more powerful a person’s social support network is, the better he or she can cope with various challenges. In this study, social support was measured by social support breadth and social support intensity, referring to the widely used Social Support Rating Scale and previous studies (23), which possessed a high universality and credibility. Specifically, social support breadth was defined as the breadth of the relationship network of individual, and was measured by the number of people who contact and communicate with each other normally. These people are relatives, friends, neighbors, etc., other than family members. Social support intensity was defined as the degree and strength of an individual social network, and was measured by the frequency of interaction with others, with the coding ranging from 1 (very infrequently) to 3 (very frequently).

Social exclusion was operated into active social exclusion and passive social exclusion, according to the measurements in the previous studies (24, 25). Active social exclusion was defined as the social break caused by their behavior and attitude against social participation, which was the result of self-active choice. Active social exclusion, in this study, was based on the question, “Are you willing to interact or contact with neighbors or other people?” The coding range was from 1 (very willing) to 5 (very unwilling). Passive social exclusion was defined as the social break that came from the outside world (15), which was not caused by their own subjective will, but by the non-subjective factors outside the individual. Passive social exclusion was based on the question “Do you feel excluded or discriminated against by others when interacting or contacting with others?” The coding range was from 1 (not at all) to 5 (severe).

### 2.3. Analysis strategy

Descriptive analyses were conducted to describe caregiver burden and other characteristics of the respondents. After adjusting for all the confounding variables, the Ordinary Least Square (OLS) methods as well as Ordered Logistic Regression methods were also employed to examine the relationship between caring load and family caregivers’ burden. Especially, the KHB method (26) was employed to examine the mediating effects of social support and social exclusion. Data analysis was implemented in Stata 15 in this study.

## 3. Results

### 3.1. Descriptive statistics

Descriptive analyses for all variables were presented in Table 1. The average burden score of family caregivers was 32.98, and caregivers in the light burden group accounted for 36.83%, followed by the little to no burden group (28.38%), moderate burden (23.74%), and heavy burden group (11.04%). Besides, the average self-assessed burden of family caregivers was 1.54. The average caring load was 1.54, and family caregivers, in extreme cases, may care for even up to 6 home-bound older adults with disabilities. As for social support, more than half of the participants were very infrequent to contact with others (59.04%), but the average scores of social support breadth were 10.92, which showed that respondents were with high levels of social support breadth, but low levels of social support intensity. In the aspect of active social exclusion, the largest proportion of the respondents was willing (42.27%) to interact with others, followed by a very willing group (31.14%). Moreover, nearly half of respondents did not feel discrimination from others in their communication (46.34%), revealing that respondents were in low levels of passive social exclusion.

**Table 1:** Descriptive statistics for all variables of the study in China, 2018 (*N* = 3,125).

Variables	Variables definition and values	Mean (S.D.)/percentage
*Dependent variables*		
Burden score [0–88]		32.98 (19.64)
Burden grade [1–4]	1 = Little to no burden	28.38%
	2 = Light burden	36.83%
	3 = Moderate burden	23.74%
	4 = Heavy burden	11.04%
Self-assessed burden [0–4]		2.67 (1.42)
*Independent variable*		
Caring load [1–6]		1.54 (0.75)
*Mediating variables*		
Social support intensity [1–3]	1 = Very infrequently	59.04%
	2 = Frequently	29.47%
	3 = Very frequently	11.49%
Social support breadth [0–30]		10.92 (9.59)
Active social exclusion [1-5]	1 = Very unwilling	2.66%
	2 = Unwilling	5.70%
	3 = General	18.24%
	4 = Willing	42.27%
	5 = Very willing	31.14%
Passive social exclusion [1–5]	1 = Not at all	46.34%
	2 = A little	19.10%
	3 = General	28.54%
	4 = Serious	2.75%
	5 = Severe	3.26%
*Control variables*		
Gender [0–1]	1 = Male	57.63%
	0 = Female	
Age [18–82]		53.49 (13.64)
Marital Status [0–1]	1 = With a spouse	71.36%
	0 = Without a spouse	
Education [1–4]	1 = Illiteracy	14.88%
	2 = Primary school	27.49%
	3 = Junior Middle school	35.33%
	4 = High school	22.30%
Family size [2–8]		3.87 (1.63)
Household income [1–5]	1 = Low income	20.67%
	2 = Middle lower income	19.04%
	3 = Middle income	21.34%
	4 = Upper income	19.04%
	5 = High income	19.90%
Community service [0–11]		1.04 (2.17)

More than half of the participants were males (57.63%) and nearly three-quarters lived without a spouse (71.36%). The average age of respondents was about 53.49. More than half of respondents (57.63%) had completed at least junior middle school education. About 40% of respondents were below middle household income status. The average number of community services was 1.04.

### 3.2. Regression results

In order to further examine the association between caring load and caregiver burden, this study used burden score to measure caregiver burden (the dependent variable), and used burden grade as well as self-assessed burden to verify the robustness of the results. Meanwhile, Ordinary Least Square (OLS) methods as well as Logistic Regression methods were used in this study, and the regression coefficients were presented in the Tables 2, 3.

**Table 2:** Results of the ordinary least square (OLS) regression in China, 2018 (*N* = 3,125).

	Burden score	Burden score	Burden score	Social support intensity	Social support breadth	Active social exclusion	Passive social exclusion
Caring load	0.058^***^	0.049^***^	0.035^**^	−0.042^**^	−0.019	−0.017	0.064^***^
Social support intensity		−0.161^***^	−0.133^***^				
Social support breadth		−0.073^***^	−0.063^***^				
Active social exclusion			0.022				
Passive social exclusion			0.237^***^				
Gender (Refer to Female)							
Male	−0.122^***^	−0.100^***^	−0.106^***^	0.082^***^	0.115^***^	0.058^***^	0.006
Age	0.088^***^	0.101^***^	0.116^***^	0.057^***^	0.051^***^	0.001	−0.073^***^
*Marital status*							
(Refer to: without a spouse)							
With a spouse	0.045^**^	0.049^***^	0.050^***^	0.008	0.044^**^	0.071^***^	−0.013
*Education (Refer to: Illiteracy)*							
Primary school	0.001	0.003	0.008	−0.001	0.035	0.009	−0.025
Junior Middle school	0.053^*^	0.058^**^	0.064^**^	0.021	0.025	0.000	−0.031
High school and above	0.087^***^	0.086^***^	0.104^***^	−0.002	−0.001	−0.052^*^	−0.071^***^
Family size	−0.041^**^	−0.004	−0.004	0.171^***^	0.128^***^	0.078^***^	−0.033
Household income							
*(Refer to: Low income)*							
Middle lower income	−0.060^***^	−0.053^**^	−0.051^**^	0.010	0.054^**^	0.018	−0.014
Middle income	0.009	0.008	0.005	−0.014	0.021	0.027	0.009
Upper income	−0.024	−0.021	−0.013	−0.006	0.056^**^	0.034	−0.036
High income	−0.051^**^	−0.044^*^	−0.038	0.015	0.060^**^	0.023	−0.032
Community service	−0.038^**^	−0.028	−0.021	0.041^**^	0.037^**^	0.039^**^	−0.039^**^
R-squared	0.036	0.076	0.129	0.041	0.048	0.026	0.017

Standard errors in parentheses; ^***^p < 0.01, ^**^p < 0.05, ^*^p < 0.1.

**Table 3:** Results of the ordinary least square (OLS) regression and logistic regression in China, 2018 (*N* = 3,125).

	Burden grade	Burden grade	Burden grade	Self-assessed burden	Self-assessed burden	Self-assessed burden
Caring load	0.0055^***^	0.0048^***^	0.0036^**^	0.046^***^	0.040^**^	0.029^*^
Social support intensity		−0.017^***^	−0.014^***^		−0.154^***^	−0.123^***^
Passive social exclusion			0.022^***^			0.192^***^
Control variables	Control	Control	Control	Control	Control	Control
Pseudo R2/R2	0.013	0.026	0.046	0.025	0.048	0.084

Standard errors in parentheses; ^***^p < 0.01, ^**^p < 0.05, ^*^p < 0.1.

The regression results in the front three columns in Table 2 showed that caring load (β = 0.058, *p* < 0.01; β = 0.049, *p* < 0.01; β = 0.035, *p* < 0.05) was significantly positively related to caregiver burden when controlling for covariates. Family caregivers with a more caring load had a higher risk of caregiver burden. In addition, with the four mediating variables gradually added into the regression model, the coefficient values of the independent variable showed a downward trend, indicating that the mediating effects might exist in the equations. Meanwhile in the control variables, male caregivers had lower burden scores compared to female caregivers. This is consistent with existing findings (27). Female caregivers tended to experience higher levels of physical and mental stressors and generally provide care for longer periods of time compared to male caregivers; Also male caregivers tended to view caregiving tasks less emotionally than female caregivers, which may help reduce caregiver stress (28, 29). Caregivers with a spouse had higher burden scores compared to caregivers without a spouse. This finding is also consistent with other studies (30). When a caregiver had a spouse, he/she had multiple roles to play as a caregiver, a spouse, and as a parent of a child, which created conflict between the roles, and increased stress in turn (31).

The regression results in the last four columns in Table 2 showed the regression results with the mediating variables as the dependent variables, indicating that the mediating effects of social support breadth and active social exclusion were not significant while the mediating effects of social support intensity and passive social exclusion were significant. Specifically, caring load (β = −0.042, *p* < 0.05) was significantly negatively related to social support intensity, caring load (β = 0.064, *p* < 0.01) was significantly positively related to passive social exclusion, when controlling for covariates. The heavier the caring load, the less the intensity of social support, and the higher passive social exclusion.

Furthermore, a robustness test was conducted to test the association between social support intensity and caregiver burden, as well as the association between passive social exclusion and caregiver burden, with the variables burden grade and self-assessed burden as the dependent variable. In addition, the regression coefficients are presented in Table 3.

The logistic regression results in the front three columns in Table 3 showed that caring load (β = 0.0055, *p* < 0.01; β = 0.0048, *p* < 0.01; β = 0.0036, *p* < 0.05) was significantly positively related to caregivers’ burden grade, indicating that the associations were also steadily significant when controlling for covariates gradually. In addition, social support intensity (β = −0.017, *p* < 0.01; β = −0.014, *p* < 0.01) was significantly negatively related to caregivers’ burden grade, passive social exclusion (β = 0.022, *p* < 0.01) was significantly positively related to caregivers’ burden grade, and the conclusion that social support intensity and passive social exclusion had partial mediating effects in the association was robust.

The regression results in the last three columns in Table 3 showed that caring load was (β = 0.046, *p* < 0.01; β = 0.040, *p* < 0.05; β = 0.029, *p* < 0.10) significantly positively related to caregivers’ self-assessed burden, indicating that the associations were also steadily significant when controlling for covariates gradually. Specifically, social support intensity (β = −0.154, *p* < 0.01; β = −0.123, *p* < 0.01) was significantly negatively related to caregivers’ self-assessed burden, passive social exclusion (β = 0.192, *p* < 0.01) was significantly positively related to caregivers’ self-assessed burden, and the conclusion that social support intensity and passive social exclusion had partial mediating effects in the association was robust.

### 3.3. Mediating effects

This study focuses on the mediating role of social support and social exclusion in the relationship between caring load and family caregiver burden. The contributions of the mediating effects are shown in Table 4.

**Table 4:** Results of mediating effects with the KHB method in China, 2018 (*N* = 3,125).

	Mediating effect of Social support intensity	Mediating effect of Passive social exclusion	Total Mediating effects
Burden score	14.11%	28.85%	37.52%
Burden grade	13.16%	27.12%	35.34%
Self-assessed burden	13.73%	28.49%	36.89%
Control variables	Control	Control	Control

The contributions of mediating effects of social support intensity were robust, probably between 13 and 15%. The contribution of social support intensity in the models with burden score as a dependent variable was14.11%; meanwhile, in the models with burden grade or self-assessed burden as dependent variables, the contribution ratios were 13.16% or 13.73%, respectively. The contributions of mediating effects of passive social exclusion were robust, probably between 27 and 29%. The contribution of passive social exclusion in the models with burden score as a dependent variable was 28.85%; meanwhile, in the models with burden grade or self-assessed burden as dependent variables, the contribution ratios were 27.12% or 28.49%, respectively. Table 4 showed the total mediating effects of social support and social exclusion were robust. In the model with burden score as a dependent variable, the total mediating effects of social support and social exclusion were 37.52%; in addition, in the models with burden grade or self-assessed burden as dependent variables, the total contribution ratios were 35.34% or 36.89%, respectively.

## 4. Discussion

In this study, the stress coping model is applied to investigate the burden of caregivers of disabled families in China, and the coping mechanism is expanded in combination with the specific cultural context of China, and social network (especially social support and social exclusion). The study found a significant positive association between the caring load and the caregiver burden, indicating that the greater the caring load is, the more serious the caregiver burden would be, consistent with previous studies (32). In the care process, the family caregiver would bear a greater burden due to the heavy and complicated caring load, as well as the potential social isolation and exhaustion (33).

It should also be noted that the role of family caregivers in caring for older adults comes from a specific Chinese cultural background——Filial Piety. China’s filial piety culture provides a cultural constraint that the adult children should provide various care services for older adults, including caring services for older adults with disabilities. Different from the concept of rights and obligations in western society, the link to maintaining the operation of the family care system for older adults in Chinese society is emotional exposure and kinship (11). Because of this inescapable duty in providing care for older adults with disabilities under Chinese traditional culture, the burden of caregivers is becoming an increasingly serious social concern in China who is experiencing a rapid population aging.

This study proved that social support (social support intensity rather than social support breadth) could significantly mediate the association between caring load and family caregiver burden, and the corresponding contribution was stable, probably between 13 and 15%. Compared with the previous literature, this study further deepened the conclusion that social support played a mediating role, and found that social support intensity rather than social support breadth played the mediating effect (34). Social support alleviates the family caregiver burden, maybe because of the power of social relations. Chinese traditional society is an “Acquaintance society,” which is characterized by a private relationship between people, people are connected through this relationship and form a network of relationships. Therefore, “Guanxi” is one of the typical words for “Acquaintance society.” Solid and sound social relations will strengthen caregivers’ social support, thereby alleviating the pressure burden of physical fatigue, psychological and emotional guilt, economic strain and social isolation or helplessness. However, the family caregivers with heavier caring loads have no time to participate in social interaction, which is the main component of social support intensity, and the decline of social support intensity would eventually lead to an increase in caregiver burden. The heavy caring load affects the ultimate caregiver burden, not by reducing the number of caregiver’s nodding acquaintances (e.g., ordinary friends or acquaintances), but by reducing the caregiver’s social contact strength (e.g., contact intensity with closer relatives and friends) (35).

This study also proved that social exclusion (passive social exclusion rather than active social exclusion) could significantly mediate the association between caring load and family caregiver burden, and the corresponding contribution was stable, probably between 27 and 29%. Social exclusion aggravates the burden of family caregivers, maybe because of social relations’ alienation, which is different from the power of social support. Family caregivers of older adults with disabilities suffer from “social exclusion caused by alienation,” including their becoming marginalized groups due to social contact, social relations, and group identity restrictions and restrictions. As a marginal group, family caregivers may be excluded from many aspects of social life. Social exclusion is defined as “social relation exclusion” when it is emphasized as the concept of “relation” (36). People being excluded from social relations could lead to “deprivation” and further limit people’s life opportunities. Those who are excluded from social relations will be socially, psychologically and even economically disadvantaged (37). To some extent, these findings have deepened the existing literature on this issue. Family caregivers who are responsible for caring for older adults with disabilities face the break and exclusion from others, which will further aggravate family caregivers’ burden (38). Meanwhile, social exclusion may not be the result of family caregivers’ willing choice but the result of passive acceptance (24).

This study further proved that social support and social exclusion contributed more than 30% to the mechanism of how the caring load affected the family caregivers’ burden, and these findings require policymakers to pay more attention to the mediation effects of social support and social exclusion, especially in the innovation of public policy on reducing family caregivers’ burden. In order to provide better care services for older adults with disabilities at home, social support and social integration should be paid more attention to, and it requires the informal social network to provide substantial, inclusive and stable support rather than exclusion or quarantine. Public policies should provide various support and assistance to these family caregivers, such as respite service, specific care skills training and support, etc., to optimize the well-being of caregivers. In addition, it is also important to develop community social organizations, carry out community activities, and cultivate community workers, so as to provide emotional assessment, spiritual and psychological support and other services for family caregivers.

There are some limitations to this study. First, although the data used in this study was based on a standardized questionnaire covering a wide range of characteristics of family caregivers, due to the vulnerability and sensitivity of family caregivers, the data collection referring to indicators of social support and social exclusion may be still not rich enough. Additionally, due to the limitations of the survey design, some relevant information about older adults with disabilities, caregivers and family is incomplete. There was a lack of survey information on the types and severity of disability of older adults with disabilities, the status of activities of daily living in the older adults with disabilities, number of family caregivers and particular roles of family caregivers, family composition and relationship quality of family members, etc. Actually, the data used in this study was cross-sectional data. Due to the cross-sectional design, it is a challenge for this study to make causal inferences. Fortunately, due to the different questions in the questionnaire point to different time points/periods, this makes the variables in this study have their own chronological orders. Thus, endogeneity could be dispelled to a certain extent. In the survey questionnaire, the caregiver burden is the current state of the respondents, however, social support (include social support breadth and social support intensity) and social exclusion (include active social exclusion and passive social exclusion) is asked about the situation of the respondents in the past year. There is a chronological relationship, for respondents, the state of social support and social exclusion in the front, the caregiver burden in the back. The chronological order of these variables strongly supported the mediation analyses and the causal inference in this study.

Despite the above potential research design challenge, this study still has its strengths and advantages, which enhance the necessity and value of this study. First, it is helpful for the readers to understand the unique pressure faced by Chinese caregivers in China, with the largest aging population on the earth. With the rapid aging of the population, the increase of care demand for older adults with disabilities in China has become increasingly prominent, and due to the traditional filial piety culture in China, the growing care burden of caregivers becomes an extremely urgent and severe concern. Meanwhile, due to the unique way of social network in China, which was deeply influenced by traditional culture (including filial piety and traditional clan networks), both social support and social exclusion under the specific cultural context may play an important mediating role between caring load and caregiver’s burden, which may be different from that under the cultural background of Western society. This study contributes significantly to the literature related to this topic. Second, this study used nationally representative data to investigate the relationship between caring load and caregiver’s burden, as well as the mediating role of social support and social exclusion, and this makes this study significantly different from previous studies using small-scale or regional data (33, 39, 40). The data (showing caregivers’ burden in China), used in this study, have the advantages of large-scale and representativeness, which could make up for the limitations of the cross-sectional nature of the data, to a certain extent. Third, to present more rigorously, this study used more cautious wording in interpreting the results, including trying to use association rather than causality to explain the relationship between some variables in this study. In the future, it is expected that longitudinal data could be used to provide support for better revealing the causal relationship between the above variables.

## 5. Conclusion

Based on nationally representative data, this study investigated the association between caring load and family caregivers’ burden in China, especially exploring the mediating effects of social support and social exclusion. The results showed that there was a significant positive association between the caring load and caregiver burden, and there were significant steady mediating effects of social support intensity and passive social exclusion on the association between caring load and family caregivers’ burden (the total contribution ratios of mediating effects was over 30%). Specifically, social support intensity and passive social exclusion played partial mediating roles, while social support breadth and active social exclusion did not, furthermore, the total mediating effects were about 35%, and the results were robust. In order to better cope with the rapidly rising aging and the corresponding rapidly expanding demand for potential care for older adults with disabilities, China’s public policy should pay more attention to the support of family caregivers, especially including the reinforcement of social support and social integration for these caregivers in communities.

## Data availability statement

The data analyzed in this study is subject to the following licenses/restrictions: the data that support the findings of this study are available from the Institute of Social Science Survey, Peking University, but restrictions apply to the availability of these data, which were used under license for the current study, and so are not publicly available. Researchers interested in these data can contact the corresponding author to process the data request.

## Ethics statement

The studies involving humans were approved by the Ministry of Civil Affairs of the People’s Republic of China. The studies were conducted in accordance with the local legislation and institutional requirements. The participants provided their written informed consent to participate in this study.

## Author contributions

HH participated in the design, review, revision and editing of the manuscript. XH participated in the discussion, revision, and editing of the manuscript. YX participated in the drafting and revision of the manuscript, and conducted data processing, and analysis. All authors have read and approved the manuscript.

## Funding

This study was funded by the National Social Science Fund of China (Grant Number: 22BRK045).

## Conflict of interest

The authors declare that the research was conducted in the absence of any commercial or financial relationships that could be construed as a potential conflict of interest.

## Publisher’s note

All claims expressed in this article are solely those of the authors and do not necessarily represent those of their affiliated organizations, or those of the publisher, the editors and the reviewers. Any product that may be evaluated in this article, or claim that may be made by its manufacturer, is not guaranteed or endorsed by the publisher.

## References

[1] World Health Organization (1980). International classification of impairments, disabilities, and handicaps: a manual of classification relating to the consequences of disease. Available at: https://apps.who.int/iris/handle/10665/41003. (Accessed February 25, 2023).

[2] Intas G Rokana V Stergiannis P Chalari E Anagnostopoulos F. Burden and sleeping disorders of family caregivers of hemodialysis patients with chronic kidney disease-end stage: a cross-sectional study. Adv Exp Med Biol. (2020) 1196:33–40. doi: 10.1007/978-3-030-32637-1_4, PMID: 32468305

[3] Su Y Chen A Jiang A Meng X Tang J. Quality of living among family caregivers of patients with senile dementia. Chin Rural Health Serv Admin. (2019) 39:370–3. [in Chinese]

[4] Zegwaard MI Aartsen MJ Grypdonck MH Cuijpers P. Differences in impact of long term caregiving for mentally ill older adults on the daily life of informal caregivers: a qualitative study. BMC Psychol. (2013) 13:103. doi: 10.1186/1471-244X-13-103, PMID: 23537066 PMC3617010

[5] Grad J Sainsbury P. Problems of caring for the mentally ill at home. Proc R Soc Med. (1966) 59:20–3. doi: 10.1177/003591576605900110, PMID: 5902369 PMC1900710

[6] George LK Gwyther LP. Caregiver Weil-being: a multidimensional examination of family caregivers of demented adults. Gerontologist. (1986) 26:253–9. doi: 10.1093/geront/26.3.253, PMID: 3721232

[7] Zarit SH Reever KE Bach-Peterson J. Relatives of the impaired elderly: correlates of feelings of burden. Gerontologist. (1980) 20:649–5. doi: 10.1093/geront/20.6.649 7203086

[8] Abu Bakar SH Weatherley R Omar N Abdullah F Mohamad Aun NS. Projecting social support needs of informal caregivers in Malaysia. Health Soc Care Community. (2014) 22:144–4. doi: 10.1111/hsc.12070, PMID: 24024495

[9] Holmes TH Rahe RH. The social readjustment rating scale. J Psychosom Res. (1967) 11:213–8. doi: 10.1016/0022-3999(67)90010-4 6059863

[10] DeLongis A Coyne JC Dakof G Folkman S Lazarus RS. Relationship of daily hassles, uplifts, and major life events to health status. Health Psychol. (1982) 1:119–6. doi: 10.1037/0278-6133.1.2.119

[11] Pan G. The issue of Chinese family. Beijing: Peking University Press (1993) [in Chinese].

[12] Raschke. Family structure, family happiness, and their effect on college students' personal and social adjustment. Fam Court Rev. (1977) 15:30–3. doi: 10.1111/j.174-1617.1977.tb01321.x

[13] Chiou CJ Chang H-Y Chen IP Wang HH. Social support and caregiving circumstances as predictors of caregiver burden in Taiwan. Arch Gerontol Geriatr. (2009) 48:419–4. doi: 10.1016/j.archger.2008.04.001, PMID: 18602706

[14] Caplan G. Mastery of stress: psychosocial aspects. Am J Psychiatry. (1981) 138:413–20. doi: 10.1176/ajp.138.4.413 7212098

[15] Giddens A Birdsall K. Sociology. Cambridge: Polity Press (2001).

[16] Udaya W Liu Y. Rethinking poverty: definition and measurement. Int Soc Sci J. (2019) 36:191–200. [in Chinese]

[17] Greenwood N Mezey G Smith R. Social exclusion in adult informal carers: a systematic narrative review of the experiences of informal carers of people with dementia and mental illness. Maturitas. (2018) 112:39–45. doi: 10.1016/j.maturitas.2018.03.011, PMID: 29704916

[18] Tabolli S Tinelli G Guarnera G Di Pietro C Sampogna F Abeni D. Measuring the health status of patients with vascular leg ulcers and the burden for their caregivers. Eur J Vasc Endovasc Surg. (2007) 34:613–8. doi: 10.1016/j.ejvs.2007.05.025, PMID: 17683953

[19] Wu F Wang Y Wang M Chen X Liu S. Analysis on the status of caregiving burden of the main caregiver on caregiving the Uygur and Kazak's older adults with disabilities. J Prev Med. (2014) 41:3539–41. [in Chinese]

[20] Wang Y Wu F Wang M Chen X. The influential factors of burden of the main caregiver on caregiving the Uygur and Kazak's older adults with disabilities. Chin J Gastroenterol. (2015) 35:4648–52. [in Chinese]

[21] Wang W Wang Y Wu F Wang M Chen X Yang X. Care burden and depression of home caregivers of older adults with disabilities in Uygur and Kazak. Chin J Health Statis. (2016) 33:941–3. [in Chinese]

[22] Limpawattana P Theeranut A Chindaprasirt J Sawanyawisuth K Pimporm J. Caregivers burden of older adults with chronic illnesses in the community: a cross-sectional study. J Community Health. (2013) 38:40–5. doi: 10.1007/s10900-012-9576-6 22689437

[23] Wang E Hu H He Y Xu Y. Can social support matter? The relationship between social support and mental health among bereaved parents in an only-child society: evidence from China. Health Soc Care Commun. (2021) 29:476–6. doi: 10.1111/hsc.13108 32701221

[24] Jin A. Summary of the theory of social refusal. Gansu Theory Res. (2004) 2:20–4. [in Chinese]

[25] Ko D Bae Y Han J. Social exclusion and switching barriers in Medicare part D choices. Sustainability. (2018) 10:2419. doi: 10.3390/su10072419

[26] Kohler U Karlson KB Holm A. Comparing coefficients of nested nonlinear probability models. Stata J. (2011) 11:420–8. doi: 10.1177/1536867X1101100306

[27] Schrank B Ebert-Vogel A Amering M Masel EK Neubauer M Watzke H. Gender differences in caregiver burden and its determinants in family members of terminally ill cancer patients. Psychooncology. (2016) 25:808–4. doi: 10.1002/pon.4005, PMID: 26477788

[28] Hsiao C-Y. Family demands, social support and caregiver burden in Taiwanese family caregivers living with mental illness: the role of family caregiver gender: caregiver gender in family caregiving. J Clin Nurs. (2010) 19:3494–03. doi: 10.1111/j.1365-2702.2010.03315.x, PMID: 20875050

[29] Schaffler-Schaden D Krutter S Seymer A Eßl-Maurer R Flamm M Osterbrink J. Caring for a relative with dementia: determinants and gender differences of caregiver burden in the rural setting. Brain Sci. (2021) 11:1511. doi: 10.3390/brainsci11111511, PMID: 34827510 PMC8615550

[30] Kim H Chang M Rose K Kim S. Predictors of caregiver burden in caregivers of individuals with dementia: predictors of caregiver burden. J Adv Nurs. (2012) 68:846–5. doi: 10.1111/j.1365-2648.2011.05787.x 21793872

[31] Pearlin LI Mullan JT Semple SJ Skaff MM. Caregiving and the stress process: an overview of concepts and their measures. Gerontologist. (1990) 30:583–4. doi: 10.1093/geront/30.5.583, PMID: 2276631

[32] Clyburn LD Stones MJ Hadjistavropoulos T Tuokko H. Predicting caregiver burden and depression in Alzheimer’s disease. J Gerontol B Psychol Sci Soc Sc*i*. (2000) 55:S2–S13. doi: 10.1093/geronb/55.1.s2 10728125

[33] Wang M Wang Y Wu F Chen X Wang W. Depression status of caregivers of older adults with disabilities in Xinjiang Uygur and Kazak. Chin Ment Health J. (2015) 29:587–2. [in Chinese]

[34] Thielemann PA Conner NE. Social support as a mediator of depression in caregivers of patients with end-stage disease. J Hosp Palliat Nurs. (2009) 11:82–90. doi: 10.1097/NJH.0b013e31819974f9

[35] Ko S-H Lee MC Baumann SL. Reducing the burden of dementia in Korea. Nurs Sci Q. (2007) 20:178–2. doi: 10.1177/0894318407299893 17442869

[36] Percy-Smith J. Policy responses to social exclusion: Towards inclusion? Buckingham: Open University Press (2000).

[37] Sen A Wang Y. On social exclusion. Comp Econ Soc Syst. (2005) 3:1–7. [in Chinese]

[38] Kitrungrote L Cohen MZ. Quality of life of family caregivers of patients with Cancer: a literature review. Oncol Nurs Forum. (2006) 33:625–2. doi: 10.1188/06.ONF.625-632 16676018

[39] Li M Yang H Gao Q Li S. Analysis of burden of family long-term caregivers for disabled elders in Jinan city and influencing factors. J Shand Univ (Health Sci). (2013) 51:109–2. [in Chinese]

[40] Liu X Yang Y Xue X Si L. Correlation between family care quality and caregiver burden of older adults with disabilities. Chin J Gerontol. (2019) 39:4081–4. [in Chinese]

